# Can Diets Be Healthy, Sustainable, and Equitable?

**DOI:** 10.1007/s13679-019-00362-0

**Published:** 2019-10-25

**Authors:** Jessica Fanzo, Claire Davis

**Affiliations:** 1 Berman Institute of Bioethics, Johns Hopkins University, Baltimore, MD USA; 2 School of Advanced International Studies, Johns Hopkins University, Washington, DC USA; 3 Bloomberg School of Public Health, Johns Hopkins University, Baltimore, MD USA

**Keywords:** Diets, Health, Sustainability, Equity, Obesity, Food Systems

## Abstract

**Purpose:**

The purpose of this study was to review the evidence on global dietary intake and trends in dietary patterns over time and to examine associations between diets and health, environment, and equity.

**Recent Findings:**

Diets now serve as a significant risk factor for the global burden of disease and death. Diet-related non-communicable disease and rising obesity are increasingly prevalent, affecting much of the global population. At the same time, the food system is producing food in ways that are not aligned with planetary health. Inequity restricts access to healthy diets and is associated with broad social determinants.

**Summary:**

Current dietary patterns are increasingly unhealthy, unsustainable, and inequitable for many populations. Multi-pronged interventions are needed to address the impacts of diets in order to improve human and planetary well-being.

## Introduction

The intersection of diets and food systems forms the foundation of successful public health outcomes and ensures human well-being. Diets, which are made up of different foods that contain both macro- and micro-nutrients and other important health-promoting properties, are born of food systems. Food systems produce, package, process, ship, and sell the food consumed around the world. They involve more than food production and ensuring those foods are available: they are central to improving food security and nutrition, ensuring social and gender equity, reducing rural poverty, promoting efficient management of natural resources, and improving the resilience of populations who rely on them for their livelihoods [1, 2].

Recent data on dietary intake and trends of dietary patterns suggest that what the world eats is no longer static or harmless. Sub-optimal diets, and the downstream metabolic effects, remain a top risk factor for the global burden of disease [3••,^4^,^5^]. Dietary trends also have increasing deleterious effects on the world’s natural resources, land availability, biodiversity, and overall ecosystems in the context of population growth and pressure [6–8]. At the same time, many people cannot access or afford a healthy diet due to various underlying social determinants, including poverty. These multiplier effects have enormous costs not only in pure economic terms but also in societal outcomes as well [1, 9–11]. An individual’s food choices have impacts that resonate far beyond themselves: diets reflect larger systemic issues that impact population health, sustainability, and justice.

This paper examines trends in dietary patterns over time and what the world’s population currently eats. The authors consider associations between diets and health, the environment, and equity and propose possible solutions to address the effects of diets.

## The Current State of Diets

### What People Are Eating Around the World

Understanding what foods people consume at all stages of life is important to demonstrate how food security and nutrition is linked to development, health, and well-being. This knowledge is also critical for shaping food system and nutrition policies to ensure they are health promoting and consumer-oriented in positive ways[12••]. Insight into dietary intake remains a challenge for researchers because of the nature of dietary surveys and data gathering methods, recall memory of those being surveyed, and geographical and representative coverage of survey data. The increasing availability of data on diets, sourcing, and costs, and the development of better metrics, survey tools, and open-access databases will allow researchers to develop a clearer picture of dietary trends and patterns[13••, 14]. As yet, important questions remain unanswered: What are people actually eating across the world? Where do people get their food from and how much do they pay, or are willing to pay, for food? What influences their dietary choices? Do health, environmental, or ethical issues factor into their decision making?

One of the key drivers of optimal nutritional status of populations is diets. The International Conference on Nutrition Rome Declaration states that “optimal diets, including traditional diets, meet nutrient requirements across all age groups and special nutrition needs. These diets avoid excessive intake of saturated fat, sugars, and sodium, essentially eliminating trans fats, among others” [15].

The WHO provides more detailed recommendations in that diets should be composed of a variety of foods that are of sufficient quantity, of high quality, and free from pathogens [16]. Sufficient quantity means that food eaten should meet energy needs in the form of calories based on the age, weight and size, sex, activity level, and overall stage of life for an individual. Quality refers to the types and varieties of foods and ingredients consumed by that individual. Foods can be grouped as healthy or unhealthy, though food classification is more complex than the simplified categories presented here. Healthy foods include whole grains, fruits, vegetables, nuts and seeds, beans and legumes, fish and seafood, and foods rich in total polyunsaturated fatty acids, omega-three fatty acids, and dietary fiber. Unhealthy foods include excessive amounts of unprocessed red meats, processed meats (cured and salted), overly processed starches, simple sugars and sugar-sweetened beverages, and foods containing high levels of saturated fat, trans fat, dietary cholesterol, and sodium [17].

The diversity of foods within food groups and across the range of food groups plays an important role in meeting optimal diets [18–20]. However, diversity does not always mean that the overall diet is healthy: a diversity of foods can be a combination of mixed foods that include foods high in trans fats, refined or simple sugars, and sodium, or overly refined, highly processed food items [21], which have a propensity to lead to obesity and diet-related non-communicable diseases (DR-NCDs) [22]. Food must also be safe to ensure that acute and chronic food-borne diseases are minimized during the production, processing, storage, transport, and distribution stages of food supply chains, as well as during storage, preparation, and cooking within households [23, 24].

Evidence has demonstrated that optimal nutrition early in life is essential to adult well-being, productivity, and human capital. However, few infants and young children consume nutritionally optimal diets, which has significant ramifications for early growth and development [25–27]. Data collected by UNICEF on infant and young child feeding practices show that 16% of children aged 6 to 24 months eat what is defined as a “minimally acceptable diet,” which is an indicator of both dietary diversity and meal frequency, along with continued breastfeeding [28]. Of children under the age of 6 months, only 41% are exclusively breastfed globally [1]. While there are differences in feeding practices between rural and urban areas and across all wealth groups for a variety of reasons, the diets of infants and young children remain inadequate in all countries [13••].

Dietary constraints, pressures, and influences continue into childhood and adolescence. The Global School-based Student Health Survey, a self-reported survey representing 83 economies, shows that among children and teens aged 13 to 17 years globally, approximately 30% do not eat fruit and 14% do not get access to and do not eat vegetables on a daily basis. However, 44% consume soda every day [13••]. Vitamin and mineral deficiencies are high in this age group in low-and middle-income countries (LMICs). Regional analysis indicates inadequate intake of iron, iodine, vitamin A, zinc, and calcium, with adolescent girls and populations in South Asia experiencing a greater risk of deficiency [29, 30].

Dietary data on adults also indicate sub-optimal patterns. Across regions, most diets are low in fruits, vegetables, whole grains, nuts and seeds, fiber, and legumes, and high in overly processed, packaged foods that can contain higher amounts of refined sugars, sodium, and unhealthy fats. In wealthy countries and among wealthy consumers, consumption of red and processed meats tends to be higher. These foods are often out of reach for poor consumers living in low-income countries for various reasons, including inadequate infrastructure of supply chains and cost [2, 20–22]. There are sub-regional exceptions to these trends: in much of Africa, legumes are highly consumed, and in some parts of Asia, vegetable consumption is still considered the mainstay of the diet. In low-income countries and places that restrict meat consumption for religious or cultural reasons, the consumption frequency of red meat (and perhaps other animal source foods) is low [8••, 13]. Likewise, dietary trends differ based on age, gender, and the overall stage of the lifecycle: healthier diets are more evident in older adults than younger adults, and in women than men [17].

### Dietary Patterns Are Changing

Profound dietary changes are occurring in concert with increased movement of people to urbanizing centers and cities; demographic changes among populations, with increased numbers of older populations in some parts of the world (Europe, Canada, the USA, Australia) and younger in others (Africa); and globalization and trade factors that influence goods and services, particularly in the food sector [31, 32]. The food systems and food environments that engender diets have become more interconnected from global to local levels, with longer, more complex food supply chains and different types of actors beyond just producers and consumers moving food in those chains [33–35]. With the enhanced interconnectedness of places and people, and the transitions witnessed with globalization and urbanization, there have been shifts in consumer purchases and preferences towards more so-called unhealthy, cheap, and convenient diets [36, 37]. This dietary shift has been associated with increasing prevalence of overweight and obesity and non-communicable disease (NCD) worldwide [38]. These dietary trends and their health outcomes are not just an issue for high-income countries (HICs): more LMICs are experiencing this shift in conjunction with a transition from undernutrition to overweight and obesity, and NCD risk [39–42].

When looking at trends from 1990 to 2013, consumption of most food groups and critical dietary components has increased across all regions of the world [2, 43]. While this rise in consumption is nuanced and regionally complex, the consumption of “unhealthy” food items has outpaced the consumption of “healthy” foods in most regions of the world [17]. The intake of whole grains, which is associated with a reduction in risk for diabetes, colorectal cancer, coronary heart disease, and stroke [44], rose substantially in Southeast Asia only. Consumption of processed meat, a risk factor for colorectal cancer, increased in all regions of the world, but so did fruit consumption. Vegetable consumption increased only in some parts of the world [45]. Sugar-sweetened beverage consumption grew in most regions, with the largest increase in North America. Reductions in sodium intake have been minimal in all regions of the world, but are consistently surpassing the global recommended intake of 2500 mg per day and currently stand at 4000 mg per day [2, 13, 43]. However, there are exceptions: some places experienced a decline in the intake of industrially produced trans fats, which occurred as a result of political commitment made to reduce trans fatty acids in the food supply. This decision reflected the very clear evidence on the adverse health effects of trans fats [6].

Many diets now contain a significant share of packaged, processed foods, such as sugar-sweetened beverages, baked goods, dairy products, processed meats, chips and crackers, cake mixes, pies, pastries, and sweets. Generally, packaged foods are industrially processed and high in salt, sugar, and saturated and trans fats [13••]. The 2018 Global Nutrition Report estimated 86% of diets do not align with the WHO healthy diet recommendations, which is largely due to the heavily processed foods in diets [13••, 46, 47]. Globally, sales of total per capita volumes of packaged food rose over 13% from 2005 to 2017 [13••]. Patterns and trends in per capita sale volumes show that Europe, North America, and Oceania purchased the highest volumes of these packaged foods between 2005 and 2017, with some stagnation or declining sales growth over that time period. Latin America and Africa are undergoing significant growth in sales of packaged foods, albeit from a lower baseline compared with North America. The number of kilocalories purchased from sugar-sweetened beverages is highest in HICs. Sales trends indicate that in many LMICs there have been modest increases in these types of beverage sales from 2009 to 2017 [13••, 48, 49].

People are increasingly eating away from home. Since 1995, the amount of overall income spent on foods eaten away from home has risen significantly in Latin America, particularly in Brazil, Chile, and Colombia [42]. Over the last 40 years, snacks eaten “on the go” have replaced main lunches and dinners in the USA [50]. This change is also apparent in Asia: in China, people who live in neighborhoods with a higher density of restaurants tend to eat breakfast and dinner away from home, a trend that is positively associated with increased overweight [51]. Portion sizes, particularly in the USA, UK, and Latin America, have significantly increased over the last few decades. These trends illustrate that the global population is cooking less and eating out more, largely due to lifestyle changes [31]. The shift to food eaten outside the home along with portion size has likely contributed to the rising obesity pandemic [52, 53].

## The consequences of diets

### Are diets healthy?

What the world eats is now considered a major risk factor of multiple forms of malnutrition and health outcomes. Sub-optimal diets, made up of unhealthy foods, are among the top risk factors globally for deaths and disability-adjusted life-years (DALYs) lost, with 11 million deaths and 255 million DALYs attributable to various dietary risk factors [3••, 4]. The percent contribution of malnutrition in all its forms (undernutrition, overweight and obesity, and dietary risks) has surpassed tobacco smoking, high blood pressure, and high fasting plasma glucose in their contributions to DALYs lost globally. In comparing countries by their socio-development income status, the combined nutrition and dietary risk presents as the top contributor, regardless of wealth status [3••, 5].

**Fig. 1:**
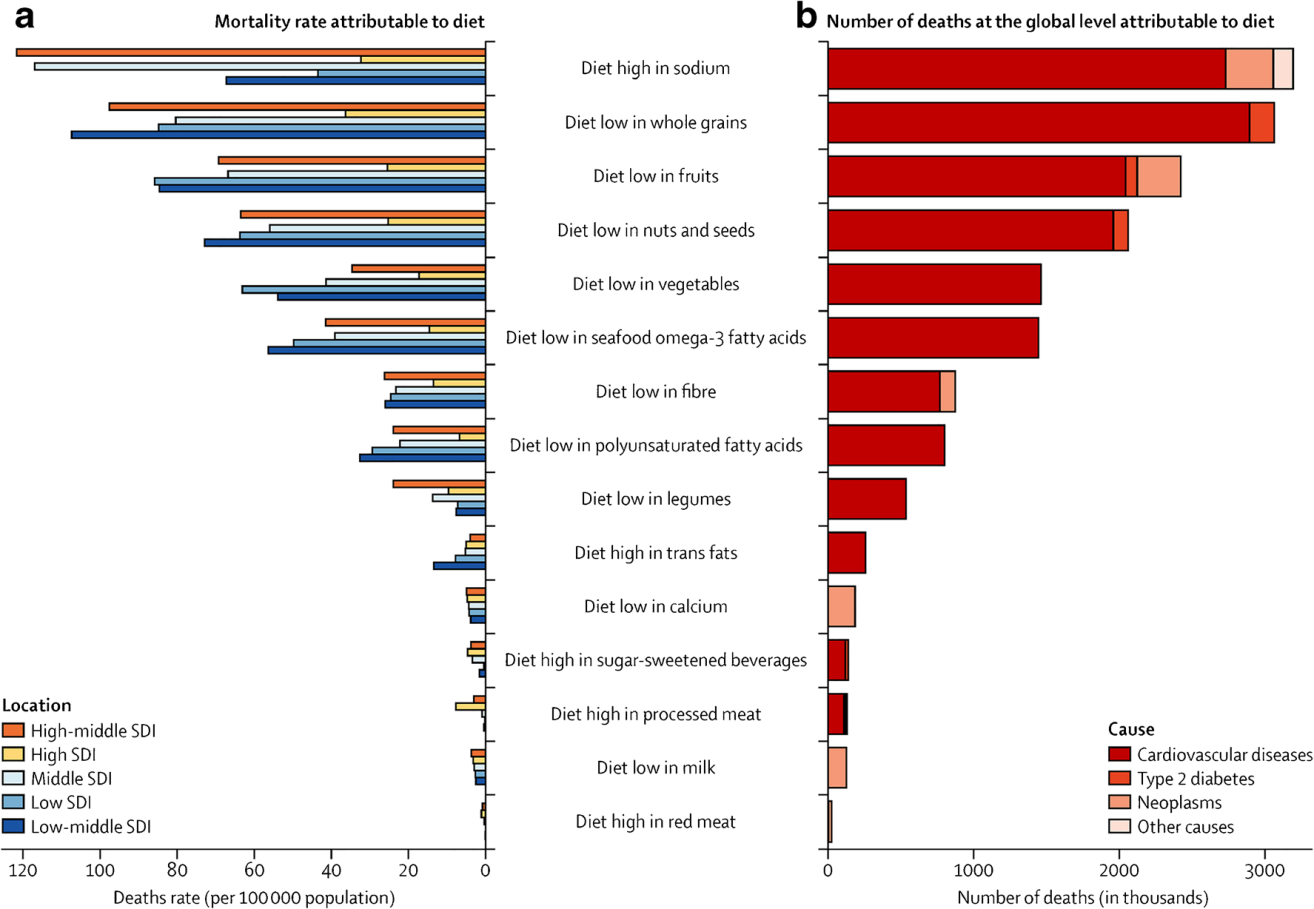
Dietary risk factor contributors to mortality, globally and across socio-development indices. Source: adapted from [5••]

Data suggest that the level of risk towards disability and death depends on the pattern of the diet and the composition of the foods that make up that diet. A recent study in the Lancet shows that globally, diets low in whole grains are the most significant dietary risk factor for deaths and DALYs. The other top five rankings consist of dietary risk factors for diets low in fruits, vegetables, nuts, and seeds, and diets high in sodium. Of the countries with high populations, high consumption of red meat, processed meat, and sugar-sweetened beverages as well as foods with high levels of trans fats were ranked lower as dietary risks for increased death and DALYs. These estimates suggest that not eating healthy foods is more detrimental to health status than eating unhealthy foods (Fig. 1b). The deaths and DALYs are mainly comprised of NCDs, including cardiovascular disease, diabetes, and cancer [5••, 8, 22].

Dietary risk varies from region to region. The main dietary risk factors across East Asia, particularly in China, Japan, and Thailand, are due to highly cured foods. Diets low in fruits and some vegetables represent the highest risk factor for sub-Saharan Africa, which suggests that value chains do not always work well on the lower part of the continent. What is not eaten is also important. Low consumption of nuts and seeds represented the most significant dietary risk factor of deaths and DALYs in Latin America [5••].

Dietary risk ranges across income status and rankings as well. Low-middle and high-middle income countries deal with the highest prevalence of diet-related deaths and DALYs (Fig. 1a), whereas HICs have the lowest exposure. With the exception of low-income countries, countries at all levels of income had low consumption of whole grains, fruit, and nuts and seeds, and high consumption of sodium as the top four dietary risk factors [5••]. The 2018 Global Nutrition Report supports the finding that all income groups consume an excessive amount of sodium, and low amounts of fruits and vegetables on a daily basis [13^••^].

Along with diets being “obesogenic” in their composition [54], obesity, as a health state itself, is a risk factor for a wide range of non-communicable diseases [55, 56]. The latest data show that two billion adults are affected by overweight and 678 million with obesity. These statistics are increasing. The prevalence of overweight adults over 18 years of age increased from 35.7% in 2010 to 38.9% in 2016. Obesity prevalence in adults also showed an increase from 11.2% in 2010 to 13.1% in 2016. Globally, women have a higher prevalence of both overweight and obesity compared with men, with some stark contrasts in key regions and countries including Latin America, North Africa, and the Middle East [13••]. It should be noted that the biological outcome of obesity is only one risk factor of NCDs and there are other social determinants along the lifecycle that influence disease outcome.

### Are diets environmentally sustainable?

Human health and environmental well-being are connected by diets. Globally, food production is the largest cause of environmental change [8••]. Food production and agriculture contribute up to 30% of all greenhouse gas (GHG) emissions, occupy 40% of available land, and use 70% of available freshwater [6, 8]. Food production is among the largest drivers of biodiversity loss, species extinction, and natural resource degradation [7, 8]. Marine systems are also overburdened, with 60% of world fish stocks fully fished and more than 30% overfished [8••].

Dietary composition significantly influences environmental outcomes. Diets high in calories, added sugars, saturated fats, processed foods, and red meats are less environmentally sustainable than healthy, plant-based diets, which are associated with reductions in GHG emissions, land use, and water use [6–8]. Unless dietary patterns change, by 2050 diets higher in refined sugars, fats, oils, and meats are likely to be major contributors to an anticipated 80% increase in agricultural GHG emissions and global land clearing [7]. Though environmental benefits from plant-based diets are variable and context specific, these diets offer promise for climate change mitigation, with modeling indicating that shifting to these diets could reduce GHG emissions by 30–55% [6–8]. Dietary composition can contribute positively to both environmental outcomes and human health.

Healthy, plant-based diets also lead to positive health outcomes for people by reducing mortality, along with obesity and DR-NCD incidence [6–8]. Plant-based diets limit meat consumption, though the levels and types of animal source foods consumed can vary depending on the specific diet (e.g., Mediterranean, vegetarian). The healthy reference diet recommended by the EAT Lancet Commission includes a low to moderate amount of seafood and poultry, and little to no red meat [8••]. Adopting healthy, plant-based diets would help avert over 10 million deaths per year, a reduction of approximately 20% [8••]. Models of different healthy, plant-based diets (Mediterranean, pescetarian, and vegetarian) show reductions in the incidence rate of type II diabetes by 16–41% and of cancer by 7–13%, as well as reduced mortality from coronary heart disease of 20–26% [7]. Environmental outcomes and nutritional needs are highly context specific; replacing animal-source foods with plant-based alternatives may be more feasible in high- and middle-income countries [6].

Diets that are optimal for both humans and the planet draw on scientific targets for food intake and planetary boundaries for food production [8••]. These diets have an appropriate caloric intake and consist of various plant-based foods, low amounts of animal source foods, unsaturated fats, and small amounts of refined grains, highly processed foods, and added sugars [8••]. Other considerations for healthy, environmentally sustainable diets include nutritional adequacy, availability and affordability, sociocultural well-being, resilience, food safety, and reductions in waste and loss [57].

The shift to healthy, environmentally sustainable diets is increasingly urgent in the face of climate change. By 2050, global GHG emissions from food production are expected to increase 80% as a result of increases in population size and dietary shifts [7]. Climate-induced changes in temperature and precipitation are expected to reduce agricultural productivity, and thus limit food availability and consumption. Higher rates of obesity are possible, as people eat less expensive, more energy-dense foods and limit their physical activity [3••]. Climate-induced changes in diets and weight could cause over 500,000 deaths by 2050, largely due to risk factors related to fruit and vegetable consumption [58].

### Are diets equitable?

Dietary equity occurs when everyone has access to a nutritious, affordable, and culturally acceptable diet [59]. Diets have consequences for nutrition, health, and well-being, and dietary inequity contributes to unequal burdens of malnutrition and disease around the world. People’s food decisions are shaped by macro-level influences, the food system itself, and personal choices [9•, 12, 59, 60]. Food and dietary decisions are also influenced by social and cultural determinants of health, which encompass a wide range of considerations related to the environments in which people live. These determinants include education, health, income and wealth, employment and working conditions, and housing and living conditions [9•, 61, 62]. Together, all of these factors intersect in complex, variable ways that determine what and how people eat and can engender inequity in their diets.

In HICs, where rates of obesity and DR-NCDs are highest, dietary inequity is closely linked to wealth: high-quality diets are associated with higher socioeconomic status, while unhealthy diets high in energy and low in nutrients are more prevalent among lower-income groups [9^•^, 10, 63]. These groups are more likely to be obese and experience higher levels of dietary-related diseases [9•, 11]. Race, education, and employment also contribute to dietary quality and obesity in HICs [9•, 10, 64].

In LMICs, the relationship between wealth, diets, and malnutrition is more complex. Obesity is more prevalent among higher socioeconomic groups [10]. Lower income groups are more likely to experience the multiple burden of malnutrition [1]. As countries develop and food environments become more obesogenic, obesity prevalence shifts from higher to lower socioeconomic levels. Women at lower socioeconomic levels are especially vulnerable to this shift. Over time, it is expected that wealthier, more highly educated populations will adapt by purchasing healthier, more expensive foods, while lower income groups will bear the burden of poor diets and obesity [65].

Around the world, dietary inequity is driven by a lack of access, availability, and affordability. Healthy, nutritious foods are increasingly more expensive than energy-dense, nutrient-poor foods, putting them out of reach for lower income populations [1, 10, 11]. In HICs, people in low-income areas are less likely to have access to grocery stores; instead, these areas are more likely to have convenience stores and fast food restaurants that offer unhealthy food options [63, 64]. In LMICs, urban populations have less access to healthy options and consume more energy-dense diets high in fat, sugar, and salt as a result [65–67]. These factors also contribute to food insecurity, which is associated with obesity and binge eating in HICs [9•, 68, 69].

Dietary inequity can contribute to multiple forms of malnutrition and carry consequences that extend across generations. In HICs, micronutrient deficiencies can co-exist with overweight and obesity, as low-cost diets can provide lots of calories without sufficient nutrients, while the double burden of malnutrition is increasingly prevalent in LMICs [10, 60]. Food insecurity can negatively affect birthweight, breastfeeding, and young child feeding practices, all of which can cause undernutrition. In turn, childhood undernutrition increases the risk for developing obesity and DR-NCDs later in life [1]. Overweight and obesity in parents can also predispose their offspring to obesity [1, 68].

## Conclusion: Moving Forward

There is not one simple solution that will automatically shift diets towards those that are healthier, more environmentally sustainable, and more equitable at the national or global scale. Rather, a range of different strategies and interventions will be necessary. To integrate these three pillars of health, environmental sustainability, and equity into diets, action must be taken by different stakeholders across different temporal and spatial scales through different entry points of the food system [70]. Interventions targeting food environments, whose composition and influence on choice are associated with poor diets and obesity, have to be included along with agricultural and food supply approaches [71, 72]. Strategies will have to involve fiscal measures, regulatory and trade interventions, industry approaches, context-specific interventions, challenges to defaults and norms of information, and consumer-focused education. These approaches must be sensitive to cultural, social, and economic context, and balance the trilateral goals of health, sustainability, and equity.

This multi-pronged approach should be one that involves the whole system, including governments, industry, and consumers. Action must start with governments, which need to govern their food systems and rebalance the scales of power [71]. According to Mozaffarian and colleagues, “no country has implemented a full range of updated, comprehensive, and evidence-informed strategies to encourage a healthier and more equitable food system” [12••]. Policy makers need to create strong regulatory and fiscal frameworks that provide guidance to those who produce the diets from our food systems. Trade and subsidy policies need to align better with those that promote healthy diets. Industry goodwill and voluntary measures are not sufficient: while some in the food and beverage industry are acting in ways that benefit public health, their efforts alone are not enough and there are still acts of transgression against public health goals [72].

The burden of change should not be solely placed on the consumer’s ability to make healthy choices, either. Public understanding of nutrition and health of diets, as well as their environmental and justice impacts, is low [73]. Governments and other food system actors generally favor healthy interventions focused on individual-level efforts [74]. Paradoxically, these interventions can worsen dietary inequities and health consequences. Food choice is not simply a personal decision: food and diets are shaped by context and driven by deep, often unseen systemic and social factors. Approaches that require a lower level of personal agency are both more effective and equitable for all [74]. Achieving the trilateral goals of health, equity, and environmental sustainability may be possible through low-agency interventions that promote healthy, environmentally sustainable diets.

A multi-pronged approach is also necessary to effect change across all environmental targets. Improvements to food production practices and reductions in food loss are needed along with governance and policy action. Changes to diets will also require long-term commitment and careful attention, as the process of setting sustainable boundaries is complex and must be refined over time [8••]. These approaches must aim for the intersection between diets, health, equity, and environmental sustainability, as not all healthy diets have low environmental impacts, and not all environmentally beneficial diets maximize human health [7].

Lastly, gaps in data need to be filled by improving the quality of dietary data, which will allow for a better understanding of their impacts on a wide-range of outcomes [14, 75, 76]. Individual-level dietary data collection and analysis need to be better standardized, as does the methodology of how these data are collected across survey tools and design. Dietary data need to be collected across a broad range of countries, with more disaggregation of socioeconomic status that takes equity issues into account. Most of the dietary data comes from high-income country data, particularly in the USA. Focusing on low- and middle-income countries is critical, as these countries will likely experience the greatest increase in obesity, yet there is little research on appropriate interventions or delivery channels [60]. The impacts of different diets also need to be considered in different local contexts on a range of environmental indicators. A more comprehensive examination of the environmental impacts of diets will help clarify life cycle assessment data of how and where food is grown, and lead to a more complete view of the relationship between diets and ecosystems.
